# A Review of the Chemical Properties, Mechanisms of Action, and Structure–Activity Relationships of Jellyfish Toxins

**DOI:** 10.3390/cimb48040350

**Published:** 2026-03-26

**Authors:** Peng Wang, Long Li, Cong Kong, Zhiyu Fu, Yunfeng Li, Hai Chi

**Affiliations:** 1 Key Laboratory of Protection and Utilization of Aquatic Germplasm Resource, Liaoning Ocean and Fisheries Science Research Institute, Dalian 116023, China; wangp@ecsf.ac.cn (P.W.); liilong1995@126.com (L.L.); hkyfzy@126.com (Z.F.); yunfengli@126.com (Y.L.); 2 East China Sea Fisheries Research Institute, Chinese Academy of Fishery Sciences, Shanghai 200090, China; kongc@ecsf.ac.cn

**Keywords:** jellyfish toxins, physicochemical properties, mechanism of action, biotransformation, structure–activity relationship

## Abstract

Jellyfish, as representatives of the phylum Cnidaria, possess venoms characterized by structurally diverse and functionally complex toxins, rendering them a central focus in cnidarian toxin research. This article presents a systematic review of the physicochemical properties of jellyfish toxins, examines their mechanisms of action from a molecular biology perspective, investigates the patterns of toxin transformation in organisms, elucidates the structure–activity relationships between structure and toxicity, introduces advancements in research on novel jellyfish toxins, and offers an outlook on future developments in this field. By integrating modern proteomic techniques, such as liquid chromatography-tandem mass spectrometry, this review provides comprehensive theoretical support for the foundational research and application development of jellyfish toxins, as well as a scientific basis for practical applications, including antivenom serum development and novel marine drug design.

## 1. Introduction

**Figure 1:**
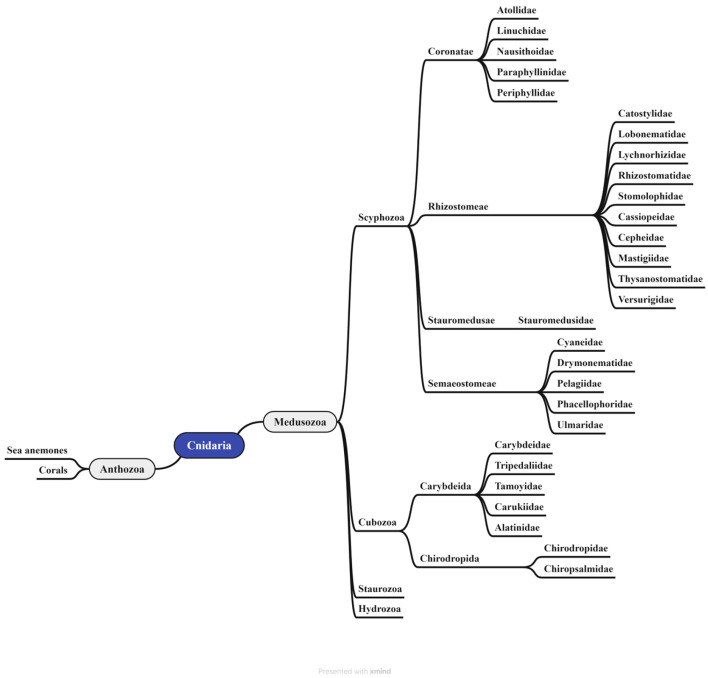
Species classification of the Cnidaria, from phylum to order. The phylum Cnidaria is divided into two subphyla: *Anthozoa* and *Medusozoa*. The subphylum *Medusozoa* is further classified into four classes, which correspond to “jellyfish” [1,2,3,4,5,6,7,8,9].

Jellyfish are members of the phylum Cnidaria, which includes over 10,000 known species. This phylum is divided into two primary lineages: *Anthozoa* (sea anemones and corals) and *Medusozoa* (jellyfish and hydras) (Figure 1). The subphylum *Medusozoa* is further classified into four classes: *Scyphozoa* (real jellyfish), *Cubozoa* (box jellyfish), *Staurozoa* (stalked jellyfish), and *Hydrozoa* (hydras) [1,2,3]. According to the World Register of Marine Species (http://www.marinespecies.org), there are currently 187 and 46 confirmed species of *Scyphozoa* and *Cubozoa*, respectively. The class *Scyphozoa* encompasses four orders: *Coronatae* (crown jellyfish), *Rhizostomeae* (root-mouth jellyfish), *Stauromedusae*, and *Semaeostomeae* (sea nettles) [4]. In contrast, the class *Cubozoa* comprises only two orders: *Carybdeida* and *Chirodropida* [5]. Santhanam. et al. has provided a more detailed taxonomic breakdown at the family level for the orders within *Scyphozoa* [6,7]: the order *Coronatae* contains 5 families (*Atollidae*, *Linuchidae*, *Nausithoidae*, *Paraphyllinidae*, *Periphyllidae*); *Rhizostomeae* includes 10 families (*Catostylidae*, *Lobonematidae*, *Lychnorhizidae*, *Rhizostomatidae*, *Stomolophidae*, *Cassiopeidae*, *Cepheidae*, *Mastigiidae*, *Thysanostomatidae*, *Versurigidae*); *Stauromedusae* is represented by a single family (*Stauromedusidae*); and *Semaeostomeae* consists of 5 families (*Cyaneidae*, *Drymonematidae*, *Pelagiidae*, *Phacellophoridae*, and *Ulmaridae*).

**Figure 2:**
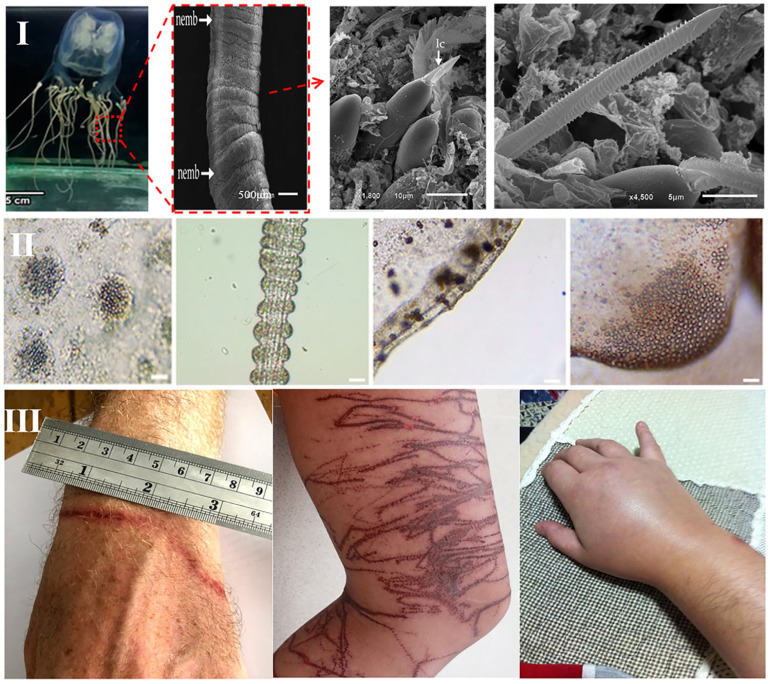
The stinging mechanism of Jellyfish and effects. (**I**) General morphology of tentacles and cnidocysts in *Cubozoans*. nemeb: nematocyst bands; lc: lancet. Reprinted from Refs. [25,26], (**II**) tentacular cnidocyst of several jellyfish species (from left to right: *Rhizostoma pulmo*, *Aurelia* sp., *Cassiopea* sp., and *Rhizostoma luteum*, 50 μm). Reprinted from Ref. [27], (**III**) *Chirodropidae* jellyfish sting wound. Reprinted from Ref. [28]. All images in the figure are taken from the cited references.

Similarly, the two principal orders within the *Cubozoa* class can be taxonomically separated at the family level. The *Carybdeida* order consists of five families: *Carybdeidae*, *Tripedaliidae*, *Tamoyidae*, *Carukiidae*, and *Alatinidae*. The *Chirodropida* order is composed of two families: *Chirodropidae* and *Chiropsalmidae* [8]. As carnivorous animals, all cnidarians possess a defining feature—highly specialized stinging cells termed cnidocytes, which are densely distributed in the tentacular epithelium [3,9] (Figure 1). Cnidoblast are the precursor cells of cnidocytes. The cnidocyst vesicle is formed from post-Golgi vesicles in the cytoplasm of nematoblasts [4]. A mature cnidocyst is a complex intracellular structure consisting of a double-walled invaginated capsule, opercular lid, and intricately coiled and frequently barbed filament [10]. Each cnidocyte precursor synthesizes a single cnidocyst, containing a toxin-loaded filament that typically measures 200–800 μm in length. Cnidocysts discharge explosively in response to mechanical or chemical stimuli. The operculum opens, and the inverted filament rapidly everts, propelling the barbed tubule at a high speed into the target. This process results in the simultaneous injection of thousands of toxin-laden tubules into the prey [11,12]. In the early stages of envenomation, jellyfish stings can induce both immediate and delayed hypersensitivity reactions, and the venom components of some jellyfish species exhibit significant immunomodulatory activity. Conversely, jellyfish-derived collagen extracts and peptides can strongly promote the release of pro-inflammatory cytokines, especially tumor necrosis factor-α (TNF-α) and interferon-γ (IFN-γ). On the other hand, protein toxins and carbohydrate-rich components (e.g., glycoproteins and polysaccharides) in nematocysts can act as antigens and trigger clinical symptoms through cellular and/or humoral immune pathways [3]. The combined neurotoxic, cytotoxic, and cardiotoxic effects of the venom can lead to systemic pathophysiology and, in severe cases, death within a few minutes [9] (Figure 2).

Jellyfish envenomation has multiple toxicological consequences due to the complex composition of their venom, which contains a wide range of bioactive chemicals, such as proteins, peptides, and low-molecular-weight substances. These constituents exhibit a wide spectrum of biological activities, including hemolytic, cytotoxic, neurotoxic, and cardiovascular toxic effects [13,14,15,16] (Table 1). Advances in current separation and purification techniques, as well as proteomic technologies, have improved the identification and characterization of these venom components. Omic and biotechnological approaches have been further integrated to form a research field known as modern venomology. Mass spectrometry imaging is a highly promising technique in modern venomics. It combines classical histology with modern mass spectrometry-based omics and enables the generation of molecular spatial distribution maps based on molecular weight. The application of methods such as LC-MS/MS has allowed the precise identification of an increasing number of toxin proteins and peptides. Consequently, the chemical nature and functional attributes of an increasing number of jellyfish toxin components have been gradually elucidated [17,18,19,20,21,22,23,24].

Contemporary research on jellyfish toxins has advanced from initial phenomenological descriptions to molecular-level investigations, including chemical structure elucidation, mechanistic studies, and structure–activity relationship (SAR) analyses. However, owing to the remarkable diversity and complexity of venom constituents, the structure-function relationships of the majority of these toxins remain poorly defined. Consequently, the development and application of novel toxin analogs are still in the early stages. Therefore, a systematic review consolidating the current knowledge on the chemical properties, mechanisms of action, and SARs of jellyfish toxins is of considerable academic importance. Such synthesis is critical for advancing foundational research and facilitating translational applications in the field. Building upon recent research findings, this review focuses on elucidating the physicochemical properties, mechanisms of action, in vivo biotransformation pathways, and structure–toxicity relationships of jellyfish toxins. Furthermore, it introduces recent progress in the study of novel toxin analogs, with the goal of serving as a comprehensive reference and framework for future investigations.

**Table 1:** Distribution of several poisonous jellyfish and signs of “stings”.

Genus/Species	Family	Geographical Distribution	Effects	References
*Physalia physails*	*Physaliidae*	Cosmopolitan, the air-water interface in the pelagic ocean	Painful stings, necrotic, neurotoxic, cardiotoxic effects	[29,30,31]
*Pelagia noctiluca*	*Pelagiidae*	Cosmopolitan, the euphotic zone, the neritic zone	Redness, immediate pain, itching, urticaria, edema, a burning sensation, vesicles, papules and/or scabs	[32,33,34]
*Aurelia aurita*	*Ulmaridae*	Global temperate and subtropical shallow coastal waters	A burning pain, oedema, erythema, urticaria, ulceration, necrosis and recurrent cutaneous eruptions	[35,36]
*Cyanea capillata*	*Cyaneidae*	Temperate marine areas of the North Pacific and North Atlantic Ocean, the euphotic zone	Edema, a burning sensation, erythema, pain, redness, wheals	[37,38,39,40]
*Alatina alata*	*Alatinidae*	Tropical and subtropical coastal waters, including the region of western Atlantic Ocean and Indo-Pacific	Immediate pain, itching, persistent skin lesions, erythematous dermatitis that may be papulovesicular, hemorrhagic, or necrotic	[41,42,43]
*Linuche unguiculata*	*Linuchidae*	Tropical and subtropical coastal waters	Stinging or erythematous reaction	[44,45]
*Chironex yamaguchii*	*Carybdeidae*	Northwestern Pacific Ocean	Severe skin pain, erythema and swelling, and systemic inflammation	[46]
*Nemopilema nomurai*	*Coronatae*	Northwestern Pacific Ocean	Skin erythema, swelling and pain	[12]
*Rhopilema nomadica*	*Rhizostomatidae*	Temperate and subtropical shallow marine waters	Mild skin pruritus or erythema and swelling	[47,48]
*Carybdea rastonii*	*Cyaneidae*	Temperate marine areas of the North Pacific and North Atlantic Oceans	Skin erythema and swelling, severe pain, nausea, and muscle spasms	[33,49]
*Rhizostoma pulmo*	*Catostylidae*	Warm water regions of the Indo-West Pacific, and the Mediterranean Sea	Skin pruritus and erythema with swelling	[27,50,51]
*Chironex fleckeri*	*Carybdeidae*	Coastal waters of northern Australia, the lower zone of sandy beaches and estuaries	Immediate pain, line or border erythema, urticaria, edema, wheals, blister, superficial necrosis and wide, ladder-like rash	[52,53,54]
*Linuche aquila*	*Linuchidae*	Tropical and subtropical coastal waters	Stinging or erythematous reaction of the skin	[25,55]

## 2. Physicochemical Characterization of Jellyfish Toxins

**Figure 3:**
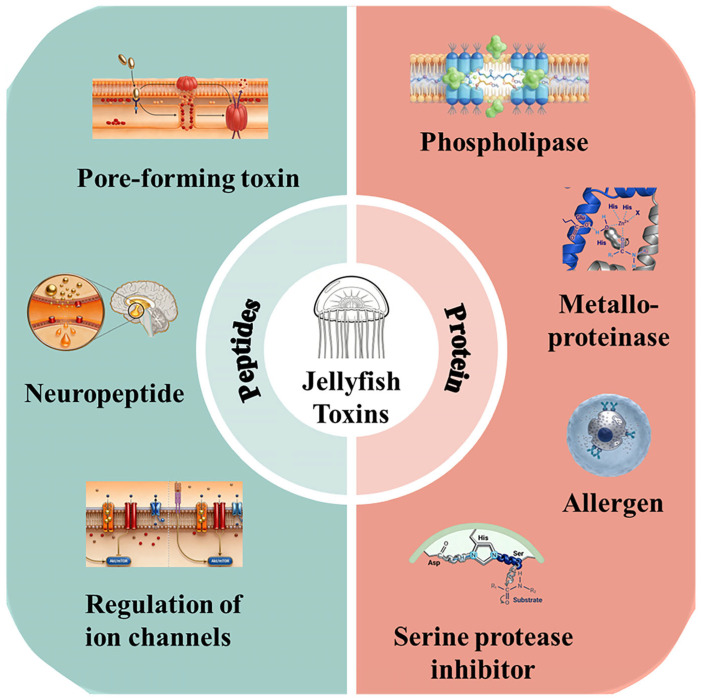
Classification of jellyfish toxins. Protein toxins are classified into four categories according to their mechanism of action: phospholipase, metalloproteinases, allergen, serine protease inhibitors; peptide toxins are classified into three categories according to their mechanism of action: pore-forming toxins, neuropeptide toxins, ion channel toxins.

The physicochemical properties of jellyfish venom vary greatly between species and toxin classes. Toxic ingredients can be further divided into two categories based on their chemical composition: protein and peptide toxins (Figure 3). The unique physicochemical profile of each toxin class, which includes features such as molecular weight, stability, and solubility, determines its biological activity and mechanism of action.

### 2.1. Protein Toxins

Proteinaceous toxins constitute the predominant and functionally central components of jellyfish venom. This category encompasses a diverse array of molecules, including phospholipase, metalloproteinases, allergens and serine protease inhibitors. The key physicochemical parameters of these proteins, such as molecular weight, isoelectric point, and structural stability, are intrinsically linked to their specific biological activities and toxicological functions [56].

The molecular weights of jellyfish protein toxins vary widely, typically ranging from 10 to 200 kDa. For instance, metalloproteinases identified in the venom of *Nemopilema nomurai* exhibit molecular weights between 30 and 60 kDa [57], whereas members of the CfTX toxin family, key toxic components in *Chironex fleckeri*, have molecular weights of 40 to 50 kDa [58]. Similarly, protease toxins derived from *Rhopilema esculentum* venom have molecular weights ranging from 25 to 75 kDa, with distinct molecular weights often correlating with specific substrate selectivity and catalytic potency [23]. Molecular size critically influences toxin pharmacokinetics; lower molecular weight proteins may diffuse more readily across biological membranes, whereas larger toxins frequently rely on specific receptor binding on the cell surface to exert their effects.

The isoelectric point (pI) represents another fundamental physicochemical factor for protein toxins. Most jellyfish protein toxins have acidic or neutral pI values. For example, the pore-forming toxin TX2 from *Chrysaora fuscescens* has a pI of approximately 5.2 [59], indicating that it is an acidic protein, whereas a serine protease inhibitor from *Chrysaora quinquecirrha* exhibits a near-neutral pI of approximately 7.1 [60]. The pI determines the net charge of a toxin under specific physiological pH conditions, thereby modulating its structural integrity, solubility, and functional interactions. Following envenomation, the local acidic microenvironment at the sting site may potentiate the activities of certain toxins. Therapeutic alkaline intervention, on the other hand, has the ability to neutralize toxicity [61], but this method still requires further investigation.

Jellyfish protein toxins are notoriously unstable and highly sensitive to thermal stress, pH extremes, and proteolytic degradation. Most toxins undergo significant or complete inactivation when exposed to temperatures above 60 °C for 10 to 15 min. Extreme pH conditions (pH < 3 or >10) can cause conformational denaturation, resulting in an irreversible loss of biological activity [62]. Furthermore, naturals proteases (e.g., trypsin and pepsin) can hydrolyze and degrade these toxin proteins, constituting a key physiological mechanism for the spontaneous alleviation of symptoms following various envenomations. Notably, certain potent toxins exhibit exceptional structural resilience. For instance, CfTX-1 from *Chironex fleckeri* demonstrates considerable structural stability under physiological conditions, which may account for the severe and long-lasting toxic reactions it induces [58].

### 2.2. Peptide Toxins

Peptide toxins represent another crucial class of bioactive constituents found in jellyfish venom. These polypeptides are typically composed of 10 to 50 amino acid residues, and possess a low molecular mass ranging from approximately 1 to 5 kDa. Despite their compact tertiary structures, they exhibit prominent and specific biological activities. This class is exemplified by the pp3 and pp11 neuropeptides obtained from the venom of *Lychnorhiza malayensis*, which have molecular masses of about 1.2 kDa and 1.5 kDa, respectively [22].

Peptide toxins have different amino acid compositions, often with a high abundance of cysteine residues. These cysteine residues generate many intramolecular disulfide bonds, which are critical for maintaining the conformational integrity and stability of the toxins. For instance, neuropeptide toxins from box jellyfish commonly contain three to four disulfide bridges in their structures. These covalent crosslinks provide structural stability and directly modulate the affinity and specificity of the toxin for its target binding sites [26]. Furthermore, certain peptide toxins undergo specific post-translational modifications, such as hydroxylation or methylation. These modifications can affect the physicochemical properties and bioactivities of the toxins. For example, the hydroxylated peptide toxins in *Aurelia coerulea* (likely caused by the increased expression of tryptophan) may significantly enhance their hydrophobicity, thereby improving their binding efficiency to cell membranes. This may be the source of toxicity in the mucus on the body surface of this jellyfish species [11].

Compared to larger protein toxins, peptide toxins often exhibit greater intrinsic stability under ambient conditions than larger protein toxins. Many enzymes retain significant activity over several days at room temperature and demonstrate considerable tolerance across a broad pH range. For example, the pp3 neuropeptide from *Lychnorhiza malayensis*, maintains over 80% of its activity across a pH range of 2 to 9, a resilience attributed to its compact, disulfide-rich scaffold [22]. Paradoxically, despite this in vitro stability, peptide toxins generally have a short in vivo half-life, typically ranging from several minutes to tens of minutes [63].

## 3. Mechanism of Action of Jellyfish Toxin

The mechanisms of action that enable jellyfish envenomation are multifaceted, and driven by a diverse repertoire of bioactive constituents. These diverse toxin components exert harmful effects by selectively targeting specific molecular locations in the organism. This targeted interference disrupts essential physiological processes, including cellular signaling, metabolic homeostasis, and membrane integrity, ultimately resulting in the observed toxicological consequences. The major mechanisms can be categorized into four principal classes based on their primary molecular targets and engagement modalities.

### 3.1. Pore-Forming Action

Pore-forming toxins (PFTs) represent one of the most common and functionally consequential components of jellyfish venom (Table 2). Their primary mechanism involves the self-assembly of transmembrane pores or channels within the lipid bilayer of the target cell membrane. This breakdown of membrane integrity disrupts ion gradients and osmotic balance, leading to the uncontrolled efflux of cytosolic contents and influx of extracellular ions, ultimately inducing cytolysis, apoptosis, or necrotic cell death [64]. Canonical examples include CfTX-A/B toxins from *Chironex fleckeri* and TX-1 toxin from *Chrysaora fuscescens* [58,59].

PFTs typically operate through a sequential, multi-stage process. First, water-soluble toxin monomers bind to the target membrane, either by directly interacting with lipid components (e.g., cholesterol) or through specific protein receptors. Subsequently, the monomers undergo conformational rearrangement, revealing hydrophobic regions that integrate into the lipid bilayer. Finally, monomers injected into the membrane oligomerize to form a stable ring-like complex that forms a functional transmembrane pore [65]. To demonstrate this mechanism, the CfTX-A toxin initially binds to membrane cholesterol. The N-terminal amphipathic helix is then inserted into the bilayer, causing approximately six monomers to assemble into a β-barrel pore with an estimated diameter of 2 nm. This pore permits the rapid efflux of K^+^ and influx of Na^+^ and Ca^2+^, resulting in cell enlargement, depolarization, and eventual rupture [58].

The cytotoxicity of PFTs is not indiscriminate; rather, it is frequently characterized by strong cellular selectivity, which is determined by the distribution of target molecules. For instance, some PFTs show a high affinity for specific glycosphingolipids found on erythrocyte membranes, thereby displaying potent hemolytic activity. Others preferentially bind to receptors enriched in vascular endothelial cells, disrupting endothelial junctions and increasing vascular permeability, resulting in clinically significant edema and hemorrhage [66].

**Table 2:** Medusozoan toxins with pore-forming toxin activity.

Name	Family	Genus/Species	References
MAC/perforin domain containing	*Rhizostomeae*	*Rhopilema esculentum*	[67]
CqTX-A	*Carybdeidae*	*Chiropsalmus quadrigatus*	[68]
CrTX-A		*Carybdea rastonii*	
TX-1	*Semaeostomeae*	*Cyanea fuscescens*	[22]
Chrysaoralin	*Pelagiidae*	*Chrysaora quinquecirrha*	
TX2	*Ulmaridae*	*Aurelia aurita*	[59]
Uncharacterised protein	*Hydridae*	*Hydra vulgaris*	

### 3.2. Enzymatic Activity

Enzymatic toxins constitute a critical class of bioactive agents found in jellyfish venom (Table 3), which exert toxicological effects by catalyzing the hydrolysis or modification of specific host substrates, thereby disrupting essential physiological pathways. Prominent enzyme families identified in these venoms include metalloproteinases and phospholipases A_2_ (PLA_2_).

Metalloproteinases are among the most prevalent enzymatic components in the human body. Multiple isoforms have been characterized in the venom of jellyfish, such as *Nemopilema nomurai* [23,67]. These zinc-dependent endopeptidases primarily target extracellular matrix (ECM) proteins, including collagen and fibronectin. Their proteolytic activity compromises tissue integrity, leading to localized damage, hemorrhage, and necrosis [69]. Certain metalloproteinases can activate pro-inflammatory cytokines, such as TNF-α and IL-6, thereby accelerating the inflammatory cascade [70]. A disintegrin and metalloproteinase (ADAM), identified in *Nemopilema nomurai* venom, degrades vascular basement membranes, increasing capillary permeability and contributing to pathological outcomes such as pulmonary edema [23].

PLA_2_ is another widely distributed enzyme in the venom of species such as *Chironex fleckeri* [20]. Its catalytic activity releases arachidonic acid and lysophosphatidylcholine from the membrane phospholipids. Arachidonic acid serves as a precursor for potent inflammatory mediators (e.g., prostaglandins and leukotrienes), whereas lysophosphatidylcholine acts as a direct cytolysin that destabilizes cell membranes and induces cell death [17].

In addition, hyaluronidase is another common venom enzyme detected in various animal venoms. Although this enzyme is non-toxic by itself, it is known as a “spreading factor” because it hydrolyzes connective tissue and promotes the diffusion of other venom components. Lee et al. detected hyaluronidase activity in the venom of *Nemopilema nomurai*, with corresponding molecular weights of 55 kDa and 95 kDa. These enzymes can directly act on and degrade extracellular matrix components, which may further lead to necrotic skin damage [69].

**Table 3:** Medusozoan toxins with enzymatic activity.

Function	Name	Family	Genus/Species	References
Phospholipase	Phospholipase A2	*Rhizostomatidae*	*Rhopilema esculentum*	[17,20]
	PLA2G12	*Nematostellidae*	*Nematostella vectensis*	
	Acidic PLA2	*Hydridae*	*Hydra vulgaris*	[71]
Metalloproteinase	Zinc metalloproteinase	*Hydridae*	*Hydra vulgaris*	
	Endothelinconverting enzyme 1/2-like			
	Carboxypeptidase D-like	*Hydridae*	*Hydra vulgaris*	[22]
	Aminopeptidase 2			
Hyaluronidase	Hyaluronidase	*Coronatae*	*Nemopilema nomurai*	[69]

### 3.3. Regulation of Ion Channels

Certain jellyfish toxins exert their effects by selectively modulating ion channel function. Toxins that specifically bind to potassium (K^+^), sodium (Na^+^), or calcium (Ca^2+^) channels disturb cellular ion homeostasis and electrophysiological signaling, which underlies a range of neurotoxic and cardiotoxic outcomes [72].

The most well-studied of these are the K^+^ channel inhibitors. For instance, the kappa-stichotoxin-Shd1a/b from *Stichodactyla haddoni* is a potent voltage-gated K+ channel blocker. Inhibition of K^+^ efflux causes membrane depolarization and disrupts neuronal repolarization and muscle contraction, contributing to cardiovascular failure [66]. Similarly, the secretory precursor Mp-332-1 from *Lychnorhiza malayensis*, which contains an ShK toxin-like domain, acts as a K^+^ channel blocker and induces prey immobilization [22].

Toxins that target Na^+^ channels typically modulate channel gating, thereby altering action potential generation. Certain jellyfish neurotoxins bind to site 3 of the voltage-gated Na^+^ channels, delaying rapid inactivation. This leads to prolonged channel opening, sustained membrane depolarization, and abnormal neuronal firing, which manifest clinically as intense pain, paresthesia, and muscle spasm [4].

Toxins that alter Ca^2+^ signaling, although less commonly documented, are also of significance. A calmodulin-like protein found in *Nemopilema nomurai* venom binds to Ca^2+^ with remarkable affinity. It can cause cellular toxicity by disrupting calcium-dependent processes, such as apoptosis and autophagy [66].

### 3.4. Immunotoxicity

Jellyfish toxins can induce immunotoxic effects through a various of interactions with the host immune system (Table 4). These interactions range from eliciting hypersensitivity reactions to directly modulating the immune cell function. A major immunopathological effect is the induction of an allergic response. Certain venom components operate as powerful allergens, causing clinical symptoms ranging from localized urticaria and pruritus to systemic anaphylaxis [52,73]. For instance, specific allergens in the venom of *Chrysaora fuscescens* can cross-link IgE antibodies bound to the surface of mast cells and basophils. This triggers degranulation and the release of preformed mediators like histamine, initiating a type I hypersensitivity reaction [59].

In addition to allergenicity, certain toxins directly impair immune cell survival and function. Toxins from certain jellyfish species have been shown to induce apoptosis in lymphocytes, reducing adaptive immune responses and potentially increasing host susceptibility to secondary infections [74]. Other venom components, on the other hand, promote pro-inflammatory properties by overactivating immune pathways. They can cause immune cells to produce excessive levels of pro-inflammatory cytokines, resulting in an unbalanced inflammatory cascade that contributes to tissue damage. For example, cytotoxic toxins from *Craspedacusta sowerbyi* trigger an amplified inflammatory response, exacerbating local tissue injury at the sting site [75].

Li et al. extracted and purified a novel polysaccharide, JSP-11, from *Rhopilema esculentum*, with a molecular weight of 1.25 × 10^6^ Da [76]. It is composed of mannose, galactose, and glucuronic acid, and exhibits significant immunomodulatory effects. JSP-11 markedly enhanced RAW 264.7 macrophage activity and promoted the release of NO, TNF-α, and IL-1β through the NF-κB, MAPKs, and PI3K/Akt signaling pathways.

**Table 4:** Jellyfish toxins with regulation of ion channel activity and immunotoxicity.

Function	Name	Family	Genus/Species	References
Regulation of ion channels	kappa-stichotoxin-Shd1 a/b	*Stichodactylidae*	*Stichodactyla haddoni*	[66]
	Calmodulin	*Rhizostomatidae*	*Rhizostoma octopus*	
	Mp-332-1		*Lychnorhiza malayensis*	[22]
Immunotoxicity	Cysteine-rich secretory protein	*Semaeostomeae*	*Chrysaora fuscescens*	[59]
	Cytotoxic toxins	*Olindiidae*	*Craspedacusta sowerbyi*	[75]
	Cell wall protein	*Hydridae*	*Hydra vulgaris*	[59]
	Polysaccharide JSP-11	*Rhizostomatidae*	*Rhopilema esculentum*	[76]

### 3.5. Enzyme Inhibitory Activity

In addition to toxins with enzymatic activity, jellyfish venom also contains toxins with enzyme inhibitory activity, which affect bodily functions by inhibiting the activity of certain enzymes in stung patients. Prakash et al. hydrolyzed the venom of *Nemopilema nomurai* with papain and isolated two novel angiotensin-converting enzyme (ACE)-inhibitory peptides: IVGRPLANG (896.48 Da) and IGDEPRHQYL (1227.65 Da) [77]. Serine protease inhibitors have been identified in various jellyfish species, including three types: Serpin (serpin superfamily) [78], Kazal-type inhibitors [79], and Kunitz-type inhibitors [23]. These factors inhibit blood clot formation.

## 4. Biotransformation of Jellyfish Toxins In Vivo

**Figure 4:**
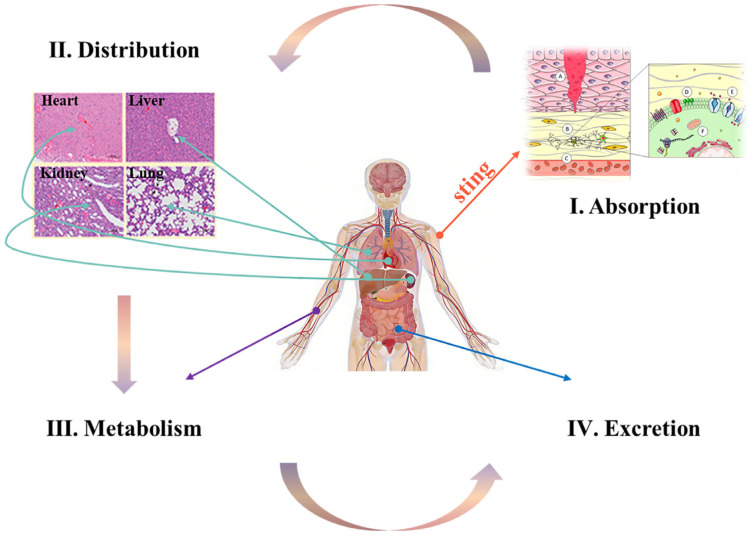
The transformation process of different jellyfish toxins in the human body. The jellyfish toxins undergo the fundamental pharmacokinetic processes of absorption (A–F, Reprinted from ref. [80]), distribution (Reprinted with permission from ref. [68]. Copyright 2020 American Chemical Society.), metabolism, and excretion (ADME).

Upon entering a biological system, jellyfish toxins undergo the fundamental pharmacokinetic processes of absorption, distribution, metabolism, and excretion (ADME) (Figure 4). This journey involves a series of enzymatic bioconversion, processes that significantly impact both the manifestation of toxicity and the in vivo clearance kinetics of these bioactive molecules.

### 4.1. Absorption

Jellyfish toxins primarily enter organisms via transdermal administration after stings. Upon nematocyst discharge, the released venom comes into direct contact with and compromises the epidermal barrier, facilitating its absorption into the systemic circulation through multiple pathways [62]. The absorption kinetics of proteinaceous and polypeptide toxins are largely contingent on the degree of damage to epidermal integrity. Envenomation typically damages the epidermal barrier, allowing toxins direct and rapid access to the dermal capillary network [81]. Conversely, certain low-molecular-weight and lipophilic toxins can passively diffuse across the intact stratum corneum [82]. The rate of transdermal absorption is strongly associated with the key physicochemical properties of the toxins. Specifically, compounds with lower molecular masses and higher lipophilicity may exhibit faster absorption kinetics.

### 4.2. Distribution

Jellyfish toxins enter into the systemic circulation and are distributed throughout the body via hemodynamic perfusion. The extent and pattern of their biodistribution are governed by three key determinants: the physicochemical properties of the toxin, its plasma protein-binding capacity, and its inherent tissue-targeting affinity [83]. The physicochemical profile of a toxin dictates its ability to cross the biological barriers. Low-molecular-weight, highly lipophilic toxins can easily cross specific barriers, such as the blood-brain and placental barriers, gaining access to the central nervous system and to fetal tissues. In contrast, high-molecular-weight, hydrophilic toxins are mostly confined to the vascular compartment and readily perfused organs like the liver and kidneys, with limited extravasation into other tissues [84].

Plasma protein binding serves as a critical pharmacokinetic modulator. The majority of proteinaceous and polypeptide toxins exhibit a high binding affinity for plasma proteins, primarily albumin and globulins. This binding traps the toxin in an inactive, macromolecular complex within the bloodstream, limiting its volume of distribution and impeding its passage across biological membranes [85].

Ultimately, specific tissue-targeting affinity underpins the organ-specific tropism and toxicodynamics of many venom components. Certain toxins possess molecular motifs that confer a high affinity for receptors or lipids that are abundant in particular tissues, leading to selective accumulation. For instance, the CfTX-1 toxin from *Chironex fleckeri* displays a pronounced affinity for cardiac tissue, binding to specific targets on cardiomyocytes and disrupting cardiac function [86]. PLA_2_ from various jellyfish species shows a high affinity for skeletal muscle, leading to localized myotoxicity [87].

### 4.3. Metabolism

Current studies have demonstrated that the major functional components of jellyfish toxins are mostly proteins or polypeptide toxins. The major metabolic mechanism of protein and polypeptide toxins is enzymatic hydrolysis. Endogenous peptidases (e.g., aminopeptidases and carboxypeptidases) degrade these toxins into amino acids or small peptide fragments, thereby eliminating their bioactivity [88]. The pp3 neuropeptide from *Lychnorhiza malayensis* is rapidly hydrolyzed to free amino acids by aminopeptidases, exhibiting a short in vivo half-life of approximately 15 min [22].

A critical consideration in toxin metabolism is the pronounced species variability. Differences in enzyme composition and activity between species lead to changes in the metabolic rates and metabolite profiles of the same toxin [89]. For example, a metalloproteinase from *Nemopilema nomurai* showed a plasma half-life of 45 min in mice but 90 min in rats, accompanied by distinct metabolite profiles in each species [90]. This diversity presents significant challenges in extrapolating toxicity data and developing broadly effective antitoxin therapies.

### 4.4. Excretion

Jellyfish toxins and their metabolites are predominantly eliminated from the body via renal excretion in urine, with biliary-fecal excretion serving as a secondary pathway. Minor amounts may also be eliminated through respiration and perspiration [62]. The physicochemical properties of the chemicals involved determine the rate and efficiency of excretion. Metabolites with high water solubility and low-molecular-weight typically undergo rapid renal clearance, whereas those with high lipophilicity and larger molecular size are excreted more slowly and frequently, require alternative pathways [82].

Renal excretion constitutes the primary elimination mechanism. Toxins and metabolites are first filtered through the glomerular capillaries. They may then be actively secreted into or passively reabsorbed by the renal tubules. Small-molecule metabolites, such as those derived from alkaloid toxins, are easily filtered and rapidly excreted in the urine [91]. In contrast, metabolites such as amino acids from degraded protein toxins can be extensively reabsorbed by the tubular epithelium for reuse, with only the remaining portion being discharged in the urine [92].

Biliary excretion plays a crucial role in the elimination of large hydrophobic compounds. These substances are actively transported from hepatocytes into the bile canaliculi, secreted into the intestine via the bile duct, and eventually expelled in feces [93]. A representative example is the high-molecular-weight CfTX-1 toxin from *Chironex fleckeri*, for which fecal excretion accounts for more than 60% of the overall clearance, highlighting the significance of the hepatobiliary pathway [86].

## 5. Structure–Toxicity Relationship

**Figure 5:**
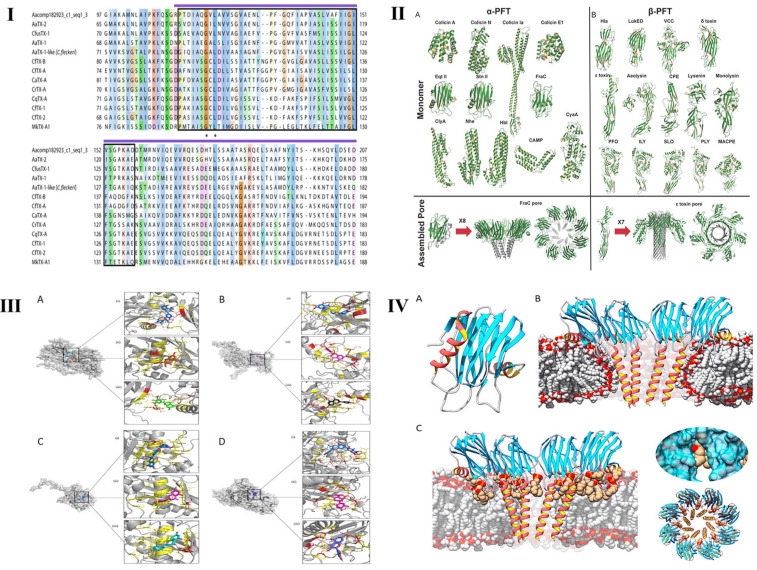
The structure–toxicity relationship of jellyfish toxins. (**I**) Partial multiple protein sequence alignment of CfusTX-1 and related jellyfish toxins, highlighting the regions of highest sequence similarity. Sequences were aligned using MUSCLE v5.3 and visualized using JalView v2.11.5.1. Amino acid residue shading is based on the Clustal protein colour scheme, with color intensity increasing as residue conservation increases from 25% to 100%. Identical residues are indicated by asterisks. Dashes represent the gaps introduced for better alignment. A predicted transmembrane spanning region (TSR1), common among jellyfish toxins, is indicated by a black outline. A purple line above the alignment corresponds to the predicted δ-endotoxin N-terminal-like domain. Reprinted from Ref. [59]. (**II**) The relationship between the secondary structure and toxicity of peptide toxins. (A): α-PFTs, upon binding to the membrane, α-helices undergo a conformational change to insert into the membrane and form membrane pore; (B): β-PFTs, monomer β-PFT first assembles in a pre-stem loop, and inserts into the membrane to form a partial β-barrel, and then combines with the other protomers to form a complete β-barrel. Reprinted from Ref. [94]. (**III**) The relationship between the tertiary structure and toxicity of peptide toxins. This is molecular docking studies of flavonoids against NnV-Mlp types and validated using AutoDock Vina v1.2.7 and PyMOL v3.1. (A), (B), (C) and (D) are the binding poses of flavonoids against Type 1/2/3/4 NnV-Mlp receptor, respectively. Reprinted from Ref. [57]. (**IV**) The relationship between the quaternary structure and toxicity of peptide toxins. (A): actinoporin monomers share a common fold: a stranded β-sandwich flanked by two short α-helixes. With regard to pore structure, two models have been proposed; (B): a tetrameric structure in which the lipid membrane adopts a toroidal shape around the pore walls; (C): an octameric lipid–protein mixed structure in which lipids (in tan color) are accommodated in pore-wall fenestrations (see inserts on the right). Reprinted from Ref. [80].

The toxicity of jellyfish venom components is governed by a well-defined structure–activity relationship (SAR). The structural hierarchy of a toxin determines its potency, target specificity, and molecular mechanisms of action. This hierarchy includes the primary structure (amino acid sequence or chemical backbone), secondary structure (e.g., α-helices and β-sheets), tertiary three-dimensional fold, and quaternary oligomeric assembly (Figure 5). A comprehensive elucidation of these structure–toxicity relationships is crucial for scientific progress, as it provides critical insights into the molecular basis of envenomation and informs the rational design of targeted antitoxin therapies.

### 5.1. Primary Structure

The bioactivity of jellyfish toxins is fundamentally determined by their structure, which directly defines their physicochemical profile and functional properties. The toxicity of protein and polypeptide toxins is influenced by their amino acid content, sequence, and post-translational changes. Residues within the active site are particularly important, and mutations at these positions can drastically change the target-binding affinity and toxic efficacy. For example, the substitution of arginine-123 with alanine in the *Chironex fleckeri* toxin CfTX-1 lowers hemolytic activity by more than 95%, as this residue is required for cholesterol binding [86].

Cysteine residues play an important role in the function of polypeptide toxins. The disulfide bridges formed are indispensable for structural integrity and are frequently integrated into the architecture of bioactive sites [95]. The pp3 neuropeptide from *Lychnorhiza malayensis*, which contains three disulfide bonds, exemplifies this: disruption of any single bond significantly diminishes activity, whereas complete reduction leads to full inactivation [22]. Furthermore, specific amino acid modifications can modulate their activities. For example, phosphorylation of a threonine residue in *Aurelia coerulea* peptide toxin enhances its binding to ion channels and increases its potency by threefold [24].

As shown in Figure 5I, a variety of pore-forming jellyfish toxins have been identified to contain a conserved transmembrane region within the N-terminal domain, sharing high sequence similarity. Their structure is homologous to the N-terminal domain of pore- forming δ-endotoxins (Cry toxins) produced by *Bacillus thuringiensis* strains. In Cry toxins, the N-terminal domain is involved in cell membrane insertion and pore formation [59].

### 5.2. Secondary Structure

Secondary structures represent the regular, hydrogen-bond-stabilized conformations within toxin polypeptides or the defined spatial arrangements of small-molecule backbones, such as α-helices, β-sheets, and β-turns in proteins. These elements are fundamental for establishing and maintaining the bioactive conformation necessary for toxicity [96].

Toxins often interact with cellular membranes through α-helices. Hydrophobic or amphipathic α-helices can anchor in the lipid bilayer and rupture membrane or generate pores. Pore-forming toxins, for instance, use an N-terminal hydrophobic α-helix to penetrate membranes and oligomerize toxin monomers into functional transmembrane pores [80]. Mutations that disrupt this helical motif drastically impair the pore-forming function. β-Sheets are extensively involved in forming the structural core of enzymatic active sites and receptor-binding interfaces. Multiple β-strands associate via hydrogen bonds to create rigid β-sheet platforms that define the architecture of the binding pockets. The catalytic site of metalloproteinases is often a cleft lined by β-sheets, with conserved residues coordinating the Zn^2+^ required for proteolytic activity [90]. β-Turns, found flexible loops connecting secondary structure elements, contribute to molecular recognition by providing conformational adaptability. These turns enable the precise alignment of key residues, enhancing the binding specificity and affinity for the target molecules.

### 5.3. Tertiary Structure

The tertiary structure refers to the overall three-dimensional folding of a toxin molecule, representing the spatial integration of its primary and secondary structural constituents. This definitive architecture directs the precise spatial arrangement of functional residues, thereby determining target recognition and binding efficacy. The active site of a toxin is typically a three-dimensional pocket or cleft formed by residues that may be distant in the linear sequence but converge in the folded structure to establish a complementary interface with the target [97]. For example, the binding pocket of a metalloproteinase is a natural tertiary structure of a protein, and its toxicity can be significantly weakened when it is bound to a competitive ligand [57,98]. Denaturation of this tertiary fold, owing to thermal stress or extreme pH, disrupts the precise spatial arrangement of these essential residues, leading to irreversible inactivation [98].

The functional surface properties of a toxin, including its electrostatic potential and hydrophobicity distribution, are directly related to its tertiary structure. These characteristics critically modulate interactions with biological membranes and influence the in vivo distribution. Pore-forming toxins demonstrate this principle, as their surface exhibits a defined amphipathic character: discrete hydrophobic patches mediate insertion into the lipid bilayer, whereas adjacent hydrophilic regions line the aqueous lumen of the assembled pore, facilitating ion conductance [80].

### 5.4. Quaternary Structure

The quaternary structure refers to the supramolecular assembly formed by non-covalent interactions between multiple toxin protomers. Many jellyfish venom components require, oligomerization before they can exhibit biological activity [62]. This is exemplified by pore-forming toxins (PFTs), which are canonical oligomerization-dependent effector proteins. Their monomers are inactive by nature and only form a functional transmembrane pore after specific oligomerization on the target membrane [80]. The CfTX-A toxin from *Chironex fleckeri*, for instance, resides in solution as an inert monomer but forms a hexameric complex on the erythrocyte membrane, gaining significant hemolytic action [99]. This oligomerization process is dynamic and regulated by factors such as local toxin concentration and membrane microenvironment composition.

Enzymatic toxins also rely on oligomerization for optimal function. For example, dimerization of a metalloproteinase from *Nemopilema nomurai*, increases its catalytic activity by approximately five-fold compared to the monomeric form (only computational simulation results) [90]. Such a quaternary structure can stabilize the active site design and increase the substrate binding affinity. In addition to directly enabling or boosting a function, oligomerization can influence the pharmacokinetic profile of toxins. The formation of higher-order complexes can confer resistance to proteolytic degradation, extend the in vivo half-life, and promote tissue accumulation, thereby exacerbating toxicological outcomes.

### 5.5. Application Value

Studies on the SAR of jellyfish toxins provide a rational framework for developing targeted therapeutic agents. By elucidating the three-dimensional architecture of toxin active sites, specific inhibitors can be rationally designed to bind and neutralize toxicity, thereby preventing pathogenic interactions with host targets [100]. Furthermore, SAR insights enable the molecular engineering of toxin derivatives for novel applications. Site-directed mutagenesis can be used to structurally modify jellyfish neuropeptide toxins, eliminating their toxicity while retaining their high affinity for neurotransmitter receptors. These synthesized peptides serve as valuable molecular tools for studying receptor function in neuroscience research [101]. Exploiting the inherent targeting specificity of toxin domains offers another promising therapeutic approach. For example, the membrane-targeting domain of a pore-forming toxin can be fused with a cytotoxic agent to create a targeted chimeric molecule. This approach facilitates the selective delivery of therapeutic payloads to tumor cells, minimizing off-target effects and enhancing anticancer efficacy [94].

## 6. Future Perspectives

While current biotechnology and analytical methods have driven substantial advances in jellyfish toxin research, numerous fundamental and translational challenges remain unresolved. Future investigations are expected to develop in more profound and integrated directions, with a concerted emphasis on bridging basic mechanistic insights and practical innovations in this field. This synergistic approach is critical for developing effective solutions to reduce the public health burden caused by jellyfish envenomation.

### 6.1. Identification of Novel Toxic Components

Despite considerable advancements, the toxin repertoire of the majority of jellyfish species remains poorly characterized. Future studies should employ a systematic venomics approach that integrates high-resolution separation techniques, high-sensitivity detection methods, and multi-omics platforms. This integrated strategy is essential for comprehensively identifying and characterizing venom components from diverse species, thereby elucidating their chemical architecture and functional repertoire. Concurrently, research on the genetic basis of toxin synthesis is essential. A better understanding of the biosynthetic pathways and regulatory processes would lay the foundation for recombinant toxin production and facilitate genetic engineering applications.

### 6.2. Elucidate the Mechanism of Action

Although the mechanisms of action of some jellyfish toxins have been characterized, the specific molecular targets and downstream signaling pathways of the vast remain elusive. Future research should prioritize elucidating these mechanisms through an integrated, multi-scale approach. High-resolution structural biology techniques, such as cryo-electron microscopy and X-ray crystallography, are essential for determining the three-dimensional architecture of toxin-target complexes, and obtaining atomic-level insights into their molecular interactions. Concurrently, these structural findings must be contextualized within a physiological framework by using cell-based assays and relevant animal models. Such studies are crucial for delineating the dynamic, multi-step processes of envenomation in vivo and identifying the key pathogenic events that lead to lethal consequences [56].

Furthermore, a critical and understudied area is the synergistic interaction among venom components. Jellyfish venom is a complex cocktail of bioactive molecules, and the overall pathophysiology is more likely caused by synergistic or potentiating interactions among its constituents rather than by the simple sum of individual toxin effects. A systematic investigation of these combinatorial interactions is fundamental for gaining a comprehensive understanding of venom toxicity and developing effective, broad-spectrum countermeasures [66].

### 6.3. New Antitoxic Drugs

The current clinical management of jellyfish envenomation remains largely supportive and symptomatic, lacking specific, mechanism-based antitoxin therapies. As result, the rational development of targeted antitoxins, guided by the elucidation of toxin SAR, represents a critical frontier in future research [4,102].

One strategic approach is to develop small-molecule inhibitors that compete with the active site of the toxin, thereby preventing it from interacting with biological targets and neutralizing its activity. Furthermore, natural products are abundant and structurally diverse resources for antitoxin development. Bioactivity-guided screening of natural compound libraries may yield novel lead molecules with low toxicity and potent inhibitory effects on venom components [4].

### 6.4. Medicinal Value of Toxins

In addition to their pathogenic effects, jellyfish toxins possess a broad spectrum of bioactive properties, indicating the significant therapeutic potential of these compounds. Certain venom components exhibit analgesic, anti-inflammatory, and antitumor activities, making them valuable lead compounds for pharmaceutical development [3]. Future research should prioritize the systematic exploration of pharmacological potential. SAR-guided optimization can also improve the bioactivity of toxin derivatives while reducing their inherent toxicity. For instance, targeted structural modifications of jellyfish neuropeptide toxins could eliminate neurotoxicity while preserving or even augmenting their analgesic potency, paving the way for novel non-opioid pain therapeutics to be developed. Previous studies have found that specific components of *Physalia physalis* and *Chironex fleckeri* exhibit inhibitory effects on KCl-induced calcium signals in small- and medium-diameter neurons [15]. Similarly, although some toxin components can permeabilize cells and damage DNA, they may also exert genoprotective effects. Through tumor-targeted molecular engineering of these toxins, their cytotoxicity can be specifically directed against cancer cells, providing a highly promising strategy for developing novel antitumor agents [98].

## 7. Conclusions

Jellyfish toxins constitute a complex and diverse biomolecular arsenal, and their pathophysiological effects are dictated by their intrinsic physicochemical properties. These properties influence the pharmacokinetic behavior of venom components, including their absorption, distribution, metabolism, and excretion, within the envenomated organism. Crucially, the SARs that underpin these toxins are key to deciphering their mechanisms of action and rationally designing targeted countermeasures. In recent years, the integration of modern separation science, proteomics, and structural biology has driven significant advances, leading to the identification of numerous venom components and gradual elucidation of their mechanisms of action.

Despite this progress, formidable challenges remain. These include the systematic cataloguing of novel toxin families, detailed mechanistic dissection of their biological interactions, and translation of this knowledge into effective, specific antitoxins. Future progress necessitates a concerted multidisciplinary strategy that utilizes innovative technologies. By systematically integrating basic research with translational applications, this field can not only reduce the public health burden of envenomation but also fully explore the latent pharmacological potential of these toxins, ultimately contributing to therapeutic innovation and improving human health.

## Data Availability

No new data were created or analyzed in this study. Data sharing is not applicable to this article.
